# Supplementary material for: A charged diatomic triple-bonded U≡N species trapped in C82 fullerene cages
Source: Nat Commun. 2022 Nov 23;13:7192. doi: 10.1038/s41467-022-34651-5 (PMC9684569; doi:10.1038/s41467-022-34651-5)
Supplement: Supplementary file 1 — Supplementery Information [file 41467_2022_34651_MOESM1_ESM.pdf]

Supplementary information

A charged diatomic triple-bonded U≡N species trapped in C_{82} fullerene cages

Qingyu Meng,[1, ⊥] Laura Abella,[2, ⊥] Yang-Rong Yao,[3] Dumitru-Claudiu Sergentu,[4] Wei Yang,[1] Xinye Liu,[1] Jiaxin Zhuang,[1] Luis Echegoyen,[5] Jochen Autschbach[2, *] and Ning Chen[1, *]

[1] College of Chemistry, Chemical Engineering and Materials Science, and State Key Laboratory of Radiation Medicine and Protection, Soochow University, Suzhou, Jiangsu 215123, P. R. China.

[2] Department of Chemistry, University at Buffalo, State University of New York. Natural Sciences Complex. Buffalo, NY 14260-3000, United States.

[3] Department of Materials Science and Engineering, University of Science and Technology of China, Hefei 230026, China.

[4] A.I. Cuza University of Iași, RA-03 Laboratory (RECENT AIR), Iași 700506, Romania.

[5] Department of Chemistry, University of Texas at El Paso, 500 W University Avenue, El Paso, Texas 79968, United States.

⊥ These authors contributed equally: Qingyu Meng, Laura Abella.

* Correspondence authors: chenning@suda.edu.cn; jochena@buffalo.edu.

Supplementary Figures

Fig. 1. HPLC chromatogram of purified UN@C_s(6)-C_{82} and UN@C_2(5)-C_{82}. 5

Fig. 2. HPLC separation of UN@C_s(6)-C_{82} 6

Fig. 3 HPLC separation of UN@C_2(5)-C_{82} 7

Fig. 4. Ball and stick representation of disordered cage and U/N sites in UN@C_2(5)-C_{82} at 100 K. 8

Fig. 5. The interaction of the major U site (a) U1 and (b) U1A with the closest cage in UN@C_2(5)-C_{82} at 100 K 8

Fig. 6. Ball and stick representation of U and N sites in UN@C_s(6)-C_{82}. at 100 K 9

Fig. 7. ADF/PBE/TZP/D3 optimized UN@C_2(5)-C_{82} and UN@C_s(6)-C_{82} geometries (blue is N and light blue is U). 9

Fig. 8. Single-crystal X-ray structure of UN@C_s(6)-C_{82}·[Ni^{II}(OEP)] measured at 100 and 185 K. 10

Fig. 9. Molecular structure of UN@C_2(5)-C_{82} measured with single crystal X-ray diffraction at variable temperatures from 100 K to 273 K. 11

Fig. 10. ZORA/PBE/TZP/D3 optimized UN@C_2(5)-C_{82} spin-doublet geometries with different orientation of the UN cluster inside the C_2-C_{82} cage. 11

Fig. 11. ZORA/PBE/TZP/D3 optimized UN@C_s(6)-C_{82} spin-doublet geometries with different orientation of the UN cluster inside the C_s-C_{82} cage. 11

Fig. 12. Partial molecular orbital (MO) diagram obtained with ZORA/PBE/TZP/D3 for the ground spin-doublet state of UN@C_2(5)-C_{82} and UN@C_s(6)-C_{82}. 12

Fig. 13. NLMO isosurfaces (±0.03 a.u.) of the carbon cage and atomic orbital %-compositions obtained from a natural bond orbital analysis of the ZORA/DFT/PBE/NBO6.0 doublet state obtained for UN@C_2(5)-C_{82}. 12

Fig. 14. NLMO isosurfaces (±0.03 a.u.) of the carbon cage and atomic orbital %-compositions obtained from a natural bond orbital analysis of the ZORA/DFT/PBE/NBO6.0 doublet state obtained for UN@C_s(6)-C_{82} 13

Fig. 15. Molecular orbital (MO) diagram obtained with ZORA/PBE/TZP/D3 for the ground spin-quartet state of UN and spin-doublet state of UN^{2+} 14

Fig. 16. Isosurface (±0.01 a.u.) of the spin density (SD) distribution for the spin-quartet state UN and spin-doublet state of UN^{2+}. 14

Fig. 17. NLMO isosurfaces (±0.03 a.u.) and atomic orbital %-compositions obtained from a natural bond orbital analysis of the ZORA/DFT/PBE/NBO6.0 doublet state obtained for UN^{2+}. 14

Fig. 18. NLMO isosurfaces (±0.03 a.u.) and atomic orbital %-compositions obtained from a natural bond orbital analysis of the ZORA/DFT/PBE/NBO6.0 quartet state obtained for UN. 15

Fig. 19. XAMS-CASPT2(-SO) Potential Energy Surface (PES) scans along the internuclear distance of UN (left) and UN^{2+} (right).. 15

Fig. 20. Shape of the sample during Raman testing. 16

Fig. 21. Vibrational normal modes of UN@C_2(5)-C_{82} assigned to the major peaks in the low-energy Raman spectra... 17

Fig. 22. Vibrational normal modes of UN@C_s(6)-C_{82} assigned to the major peaks in the low-energy Raman spectra... 17

Fig. 23. Single-crystal X-ray structure of UN@C_s(6)-C_{82}·[Ni^{II}(OEP)] measured at 100 to 273 K.. 17

Fig. 24. X-Band EPR spectrum of UN@C_2(5)-C_{82}. 18

Fig. 25. ORTEP-style illustration with probability ellipsoids for samples................. 19

Supplementary Tables

Table 1. The distance between U1 and C_{cage} in UN@C_2(5)-C_{82} at 100 K 20

Table 2. The distance between U1 and C_{cage} in UN@C_s(6)-C_{82} at 100 K.................. 20

Table 3. Adiabatic spin-state relative energies (ΔE, kcal·mol^{-1}), U Mulliken Spin Populations (MSP) and structural parameters (distances in Å) for UN@C_{82}............. 20

Table 4. Metal site occupancy in UN@C_s(6)-C_{82} as a function of temperatures........ 21

Table 5. Metal site occupancy in UN@C_2(5)-C_{82} as a function of temperatures. 21

Table 6. Crystal data of UN@C_s(6)-C_{82}.. 22

Table 7. Crystal data of UN@C_2(5)-C_{82} 23

Table 8. Adiabatic spin-state relative energies (ΔE, kcal·mol^{-1}) and U Mulliken Spin Populations (MSP) for UN@C_2(5)-C_{82} and UN@C_s(6)-C_{82} using different functionals: PBE, BP86, PBE0 and B3LYP 24

Table 9. Comparison of the calculated N-U distance (in Å) using different functionals: PBE, BP86, PBE0 and B3LYP, vs. the experimental value for UN@C_2(5)-C_{82} and UN@C_s(6)-C_{82}. 24

Table 10. Adiabatic spin-state relative energies (ΔE, kcal·mol^{-1}) and U Mulliken Spin Populations (MSP) for UN@C_2(5)-C_{82} and UN@C_s(6)-C_{82} using different software: ADF vs. G16. 24

Table 11. Comparison of the calculated N-U distance (in Å) using ADF vs. G16. The data are compared to the corresponding experimental value for UN@C_2(5)-C_{82} and UN@C_s(6)-C_{82}. 24

Table 12. Relative energies (in kcal·mol^{-1}) and structural parameters (distances in Å) of UN, obtained with ZORA/PBE/TZP/D3 for optimized UN@C_2(5)-C_{82} spin-doublet geometries with different orientation of the UN cluster inside the C_2-C_{82} cage 25

Table 13. Relative energies (in kcal·mol^{-1}) and structural parameters (distances in Å) of UN, obtained with ZORA/PBE/TZP/D3 for optimized UN@C_s(6)-C_{82} spin-doublet geometries with different orientation of the UN cluster inside the C_s(6)-C_{82} cage 25

Table 14. U-N distance in UN@C_s(6)-C_{82} as a function of temperatures. 25

Table 15. U-N distance in UN@C_2(5)-C_{82} at 100 K. 25

Table 16. Low-energy electronic states of UN at r_{eq} = 1.756 Å from wavefunction calculations. 26

Table 17. Low-energy electronic states of UN^{2+} at r_{eq} = 1.707 Å from wavefunction calculations. 26

Table 18. xyz coordinates of UN@C_2(5)-C_{82} 27

Table 19. xyz coordinates of UN@C_s(6)-C_{82} 28

Supplementary Figures

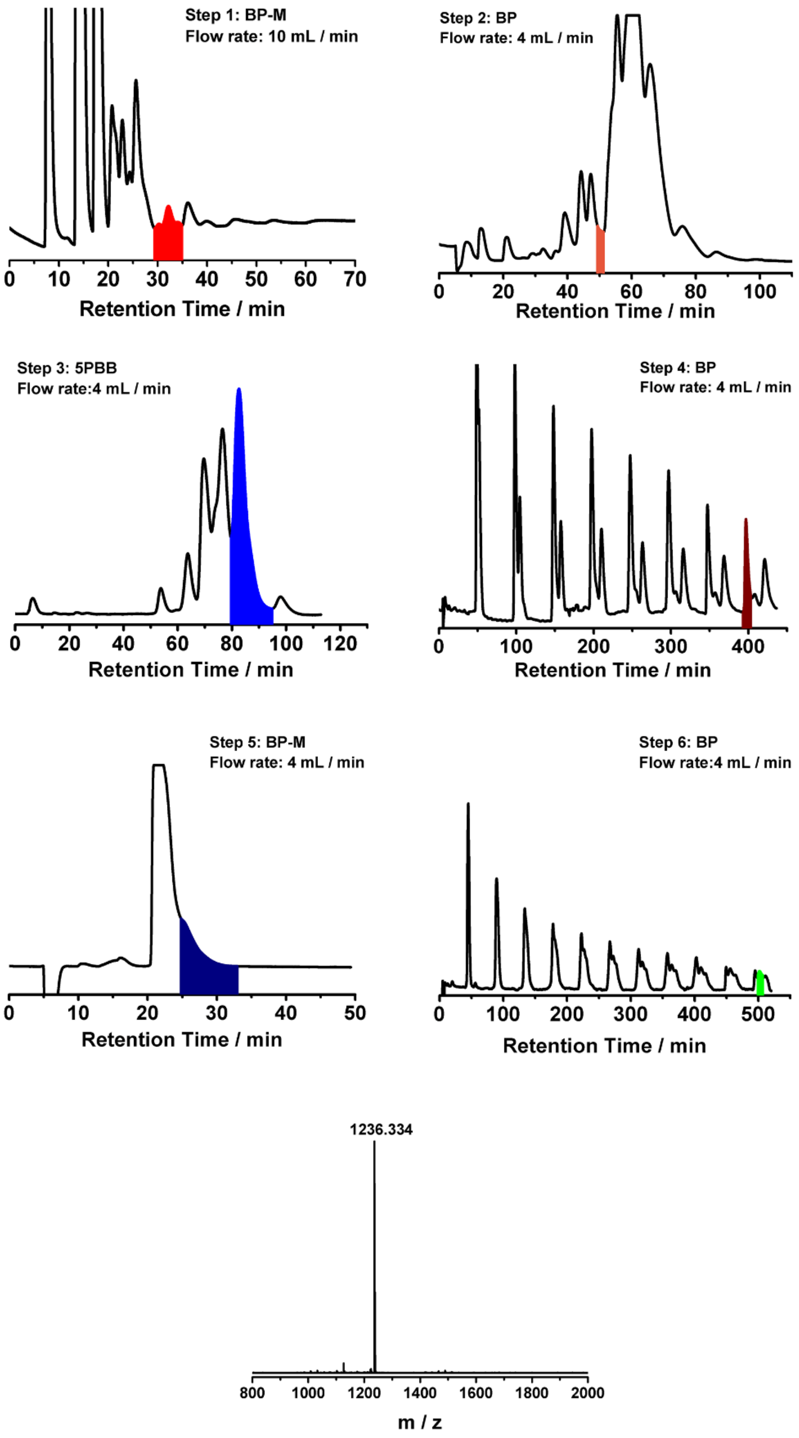

Supplementary Fig. 1. HPLC profiles showing the separation procedures of UN@C_s(6)-C_{82} and the pure sample's MALDI-TOF mass spectrum. Source data are provided as a Source Data file.

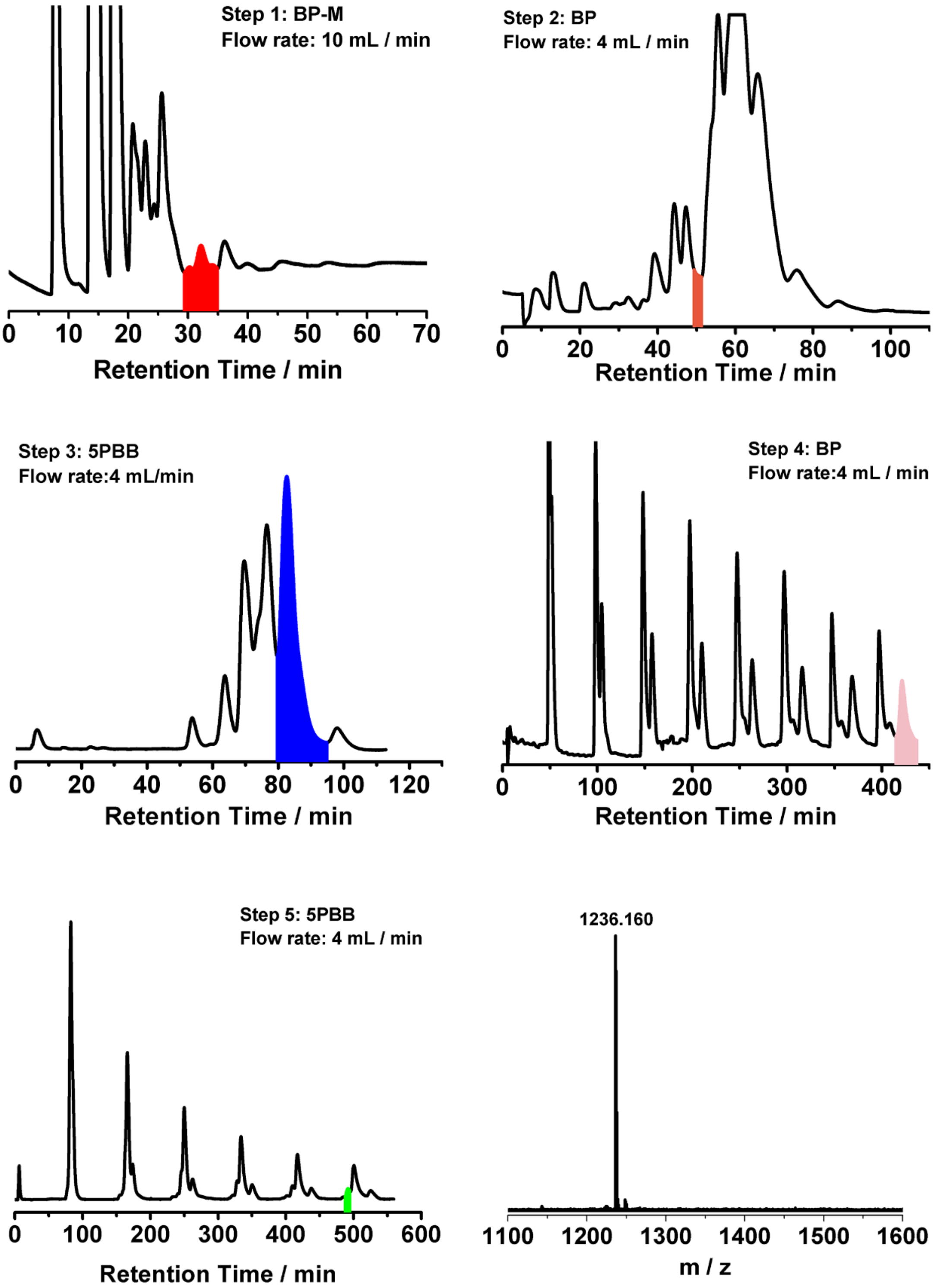

Supplementary Fig. 2. HPLC profiles showing the separation procedures of UN@C_2(5)-C_{82} and the pure sample's MALDI-TOF mass spectrum. Source data are provided as a Source Data file.

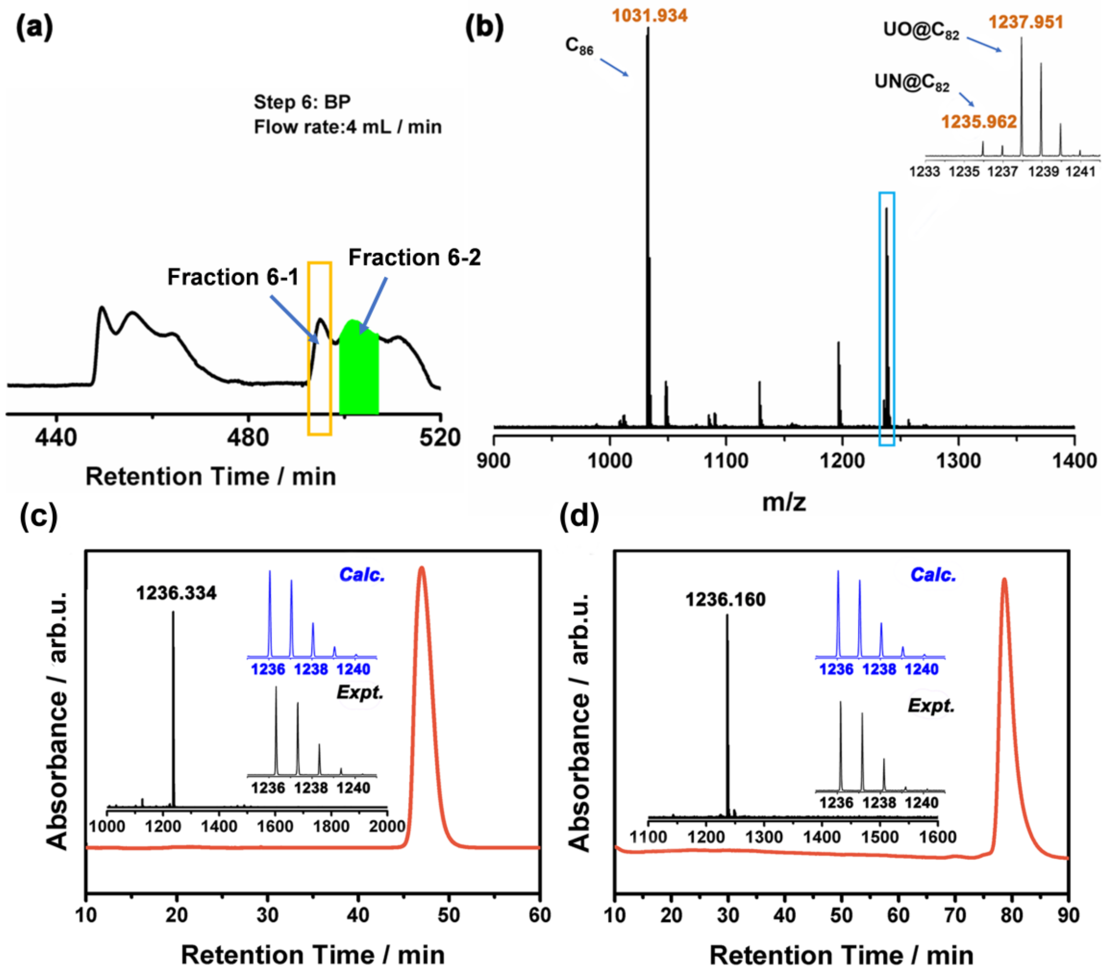

Supplementary Fig. 3. (a) Partial magnification of the sixth step of chromatographic separation of UN@C_s(6)-C_{82}. The fractions marked in green are UN@C_s(6)-C_{82}. (b) Mass spectra of fraction 6-2. The inset is an enlarged view of the mass spectra at the position marked by the blue square. The complete chromatographic separation HPLC profiles and corresponding mass spectra are shown in **Supplementary Fig. 1**. HPLC chromatograms of purified UN@C_s(6)-C_{82} and UN@C_2(5)-C_{82}. (c) UN@C_s(6)-C_{82} on a Buckyprep column and (d) UN@C_2(5)-C_{82} on a 5PBB column with toluene as the eluent. HPLC conditions, λ = 310 nm; flow rate, 4 mL/min. The insets show the positive-ion mode MALDI-TOF mass spectra and expansions of the corresponding experimental isotopic distributions of the compound in comparison with their calculated values. Source data are provided as a Source Data file.

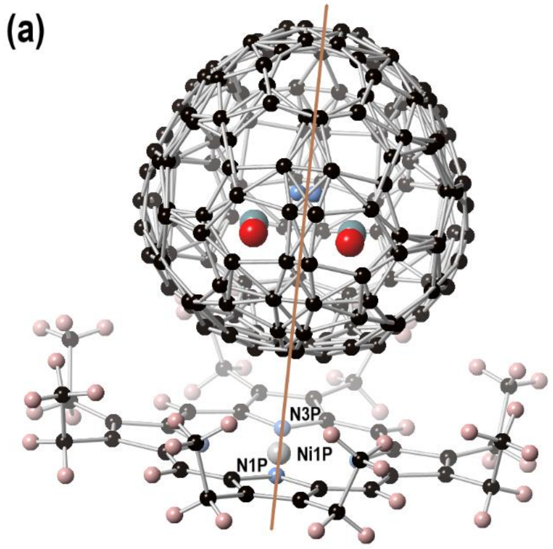

Supplementary Fig. 4. (a) Ball and stick representation of disordered cage and U/N sites in UN@C_2(5)-C_{82} at 100 K. (b) Drawing of all the U, N disordered sites, two disordered sites with fractional occupancies of 0.5 for N1 and N1A (which is generated from N1 atom by mirror plane of the crystal) atom, and four sites for U are presented, two disordered sites with fractional occupancies of 0.312 and 0.188 for U1 and U2, respectively. Another half of the U disordered sites (U1A, U2A, respectively) could be generated by mirror plane of the crystal which is vertically perpendicular to the paper plane.

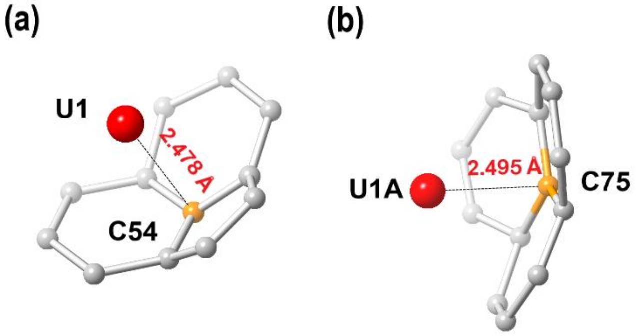

Supplementary Fig. 5. The interaction of the major U site (a) U1 and (b) U1A with the closest cage in UN@C_2(5)-C_{82} at 100 K.

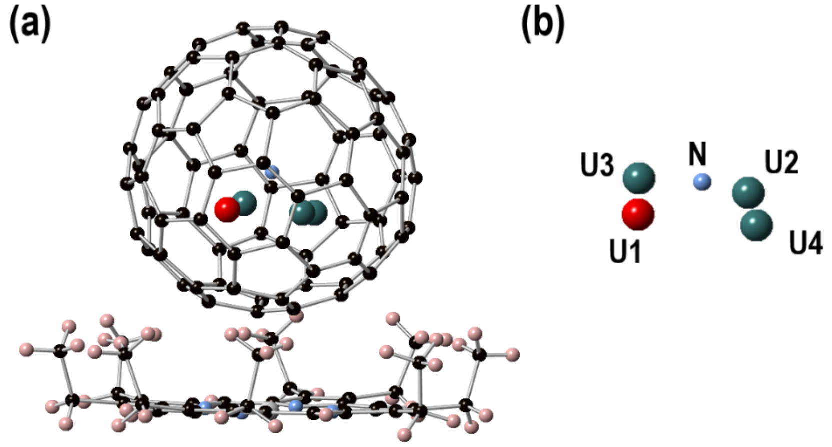

Supplementary Fig. 6. (a) Ball and stick representation of U sites and N sites in UN@C_s(6)-C_{82} at 100 K. (b) Drawing of all the U, N disordered sites, four disordered sites with fractional occupancies of 0.6442, 0.1903, 0.1087 and 0.0566 for U1, U2, U3 and U4 respectively.

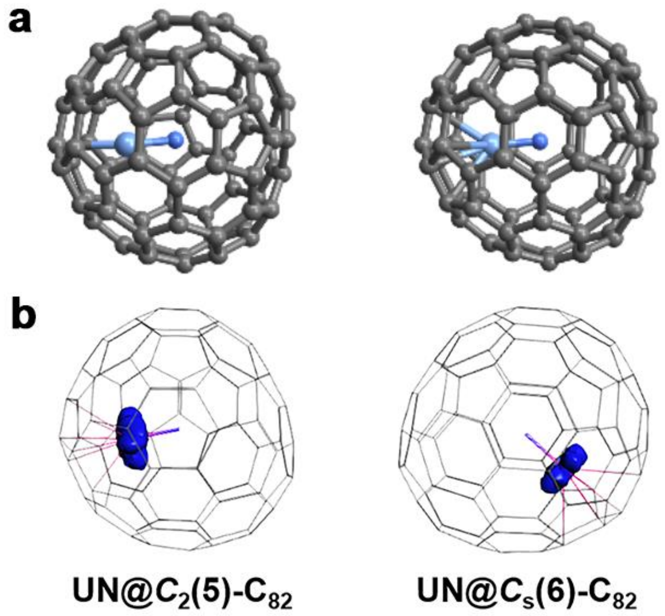

Supplementary Fig. 7. (a) ADF/PBE/TZP/D3 optimized UN@C_2(5)-C_{82} and UN@C_s(6)-C_{82} geometries (blue is N and light blue is U). (b) Isosurfaces (±0.01 au) of the calculated spin density for the optimized spin-doublet ground state structures of UN@C_2(5)-C_{82} (left) and UN@C_s(6)-C_{82} (right).

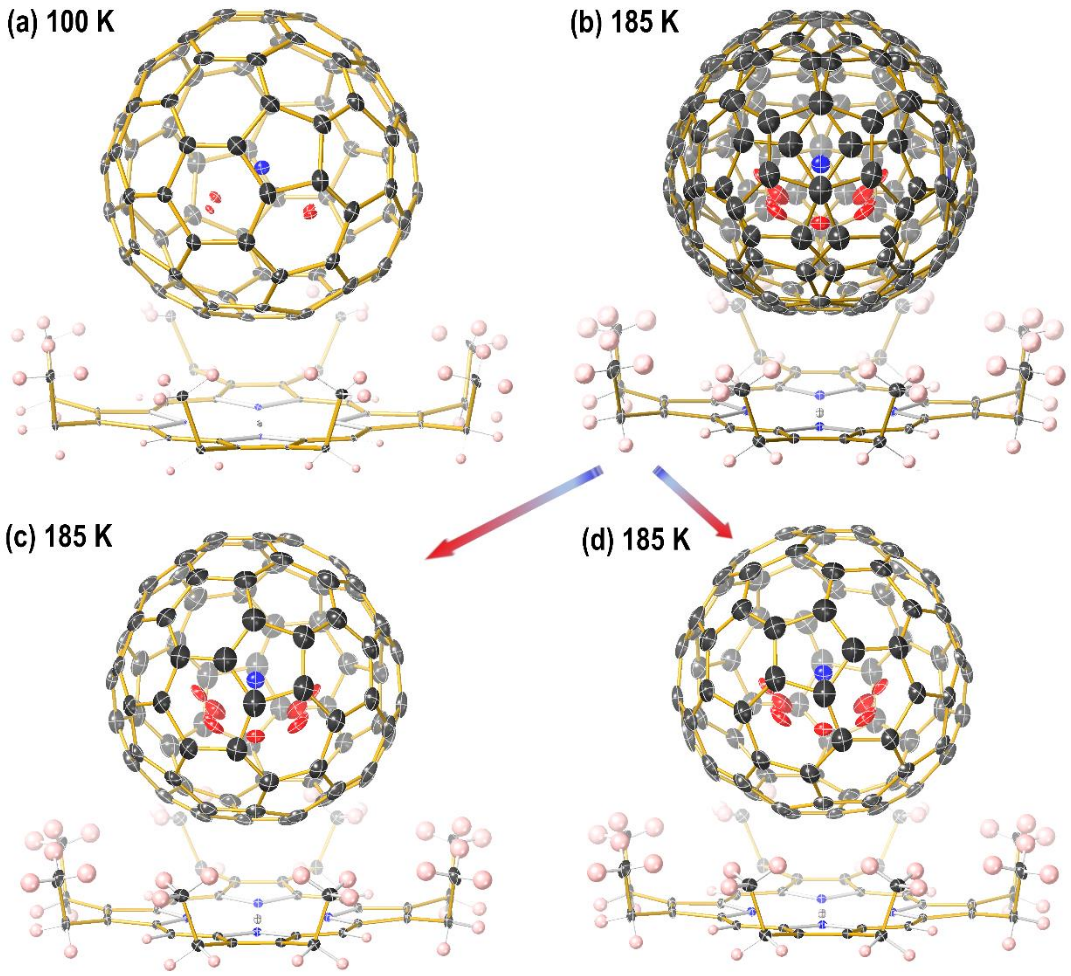

Supplementary Fig. 8. Single-crystal X-ray structure of UN@C_s(6)-C_{82}·[Ni^{II}(OEP)] measured at 100 and 185 K. Solvent molecules are omitted for clarity. The displacement parameters are shown at the 20% probability level. Color code: black for carbon, red for U, blue for N, pink for H, and grey for Ni. (a) The structure measured at 100 K is placed here for comparison. (b) The structure measured at 185 K shown with both orientations. (c, d) The two orientations of the structure measured at 185 K are split to view the structure in higher clarity.

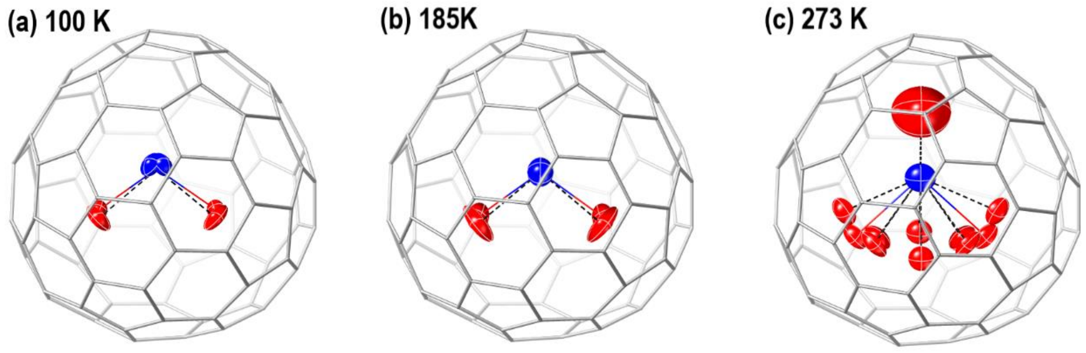

Supplementary Fig. 9. Molecular structure of UN@C_2(5)-C_{82} measured with single crystal X-ray diffraction at 100 K(**a**), 185K(**b**) and 273 K(**c**). The displacement parameters are shown at the 20 % probability level for the encapsulated UN cluster. The structures are drawn from the chosen specific direction of the crystal to compare the dynamics of the UN@C_2(5)-C_{82}. Color code: blue for N, and red for U.

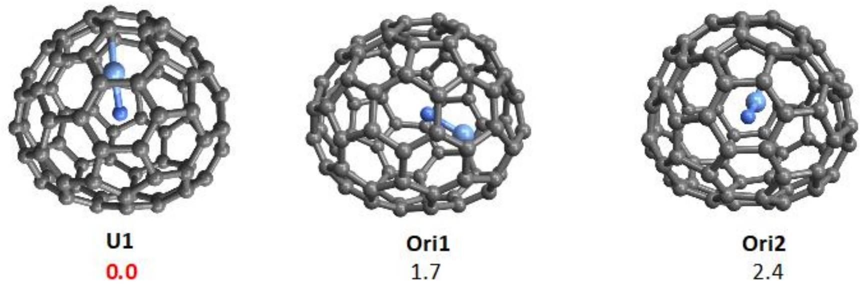

Supplementary Fig. 10. ZORA/PBE/TZP/D3 optimized UN@C_2(5)-C_{82} spin-doublet geometries with different orientation of the UN cluster inside the C_2-C_{82} cage. Relative energies in kcal·mol^{-1} are indicated below each orientation (Ori). U1 cage is the geometry optimized from the crystallographic data with the U position of U1.

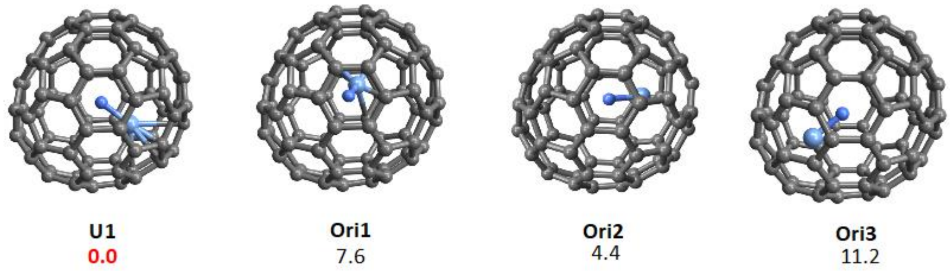

Supplementary Fig. 11. ZORA/PBE/TZP/D3 optimized UN@C_s(6)-C_{82} spin-doublet geometries with different orientation of the UN cluster inside the C_s-C_{82} cage. Relative energies in kcal·mol^{-1} are indicated below each orientation (Ori). U1 cage is the geometry optimized from the crystallographic data with the U position of U1.

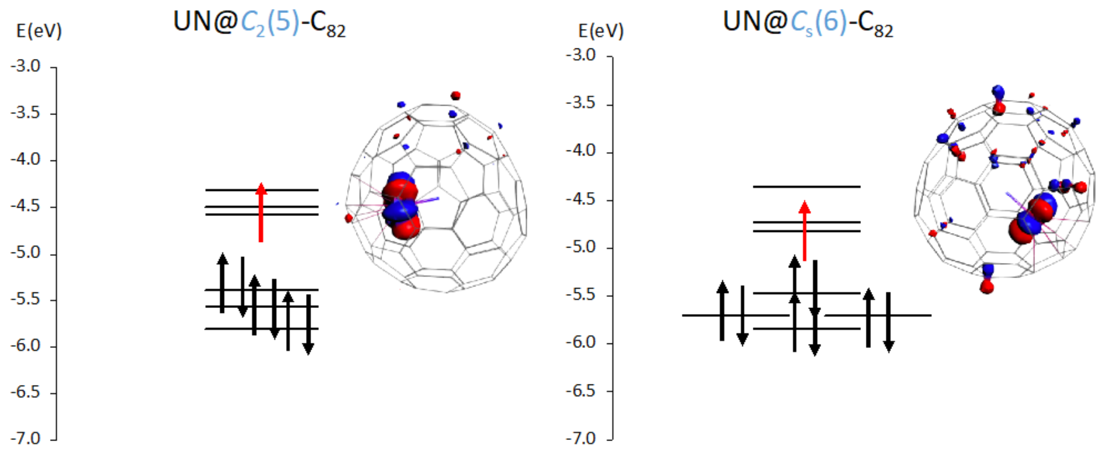

Supplementary Fig. 12. Partial molecular orbital (MO) diagram obtained with ZORA/PBE/TZP for the ground spin-doublet state of UN@C_2(5)-C_{82} (left) and UN@C_s(6)-C_{82} (right). The singly-occupied molecular orbital (for α-spin) is drawn in red and the associated MO isosurface (±0.04 a.u.) is shown on the side.

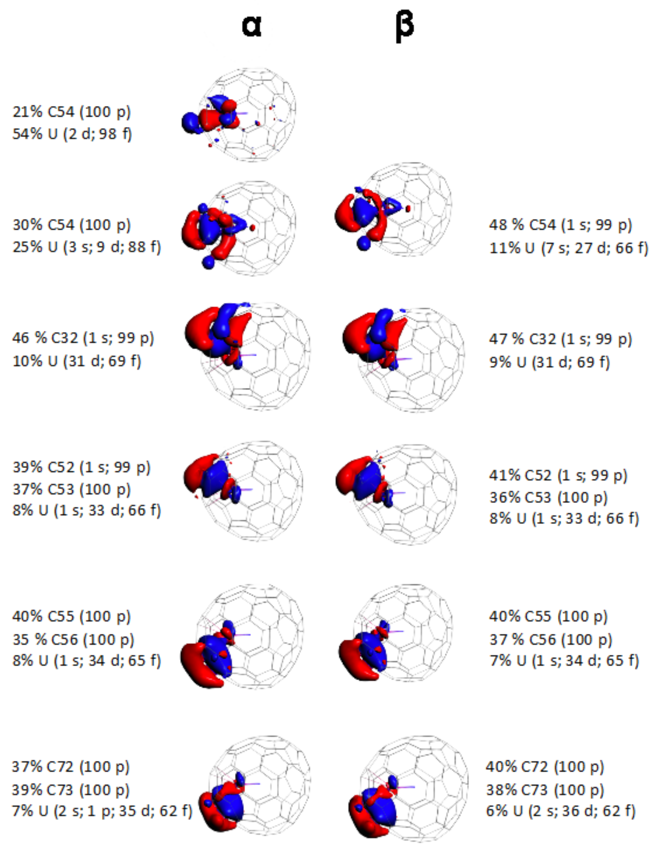

Supplementary Fig. 13. NLMO isosurfaces (±0.03 a.u.) of the carbon cage and atomic orbital %-compositions obtained from a natural bond orbital analysis of the ZORA/DFT/PBE doublet state obtained for UN@C_2(5)-C_{82}. Alpha (α)- and beta (β)-spins are plotted separately.

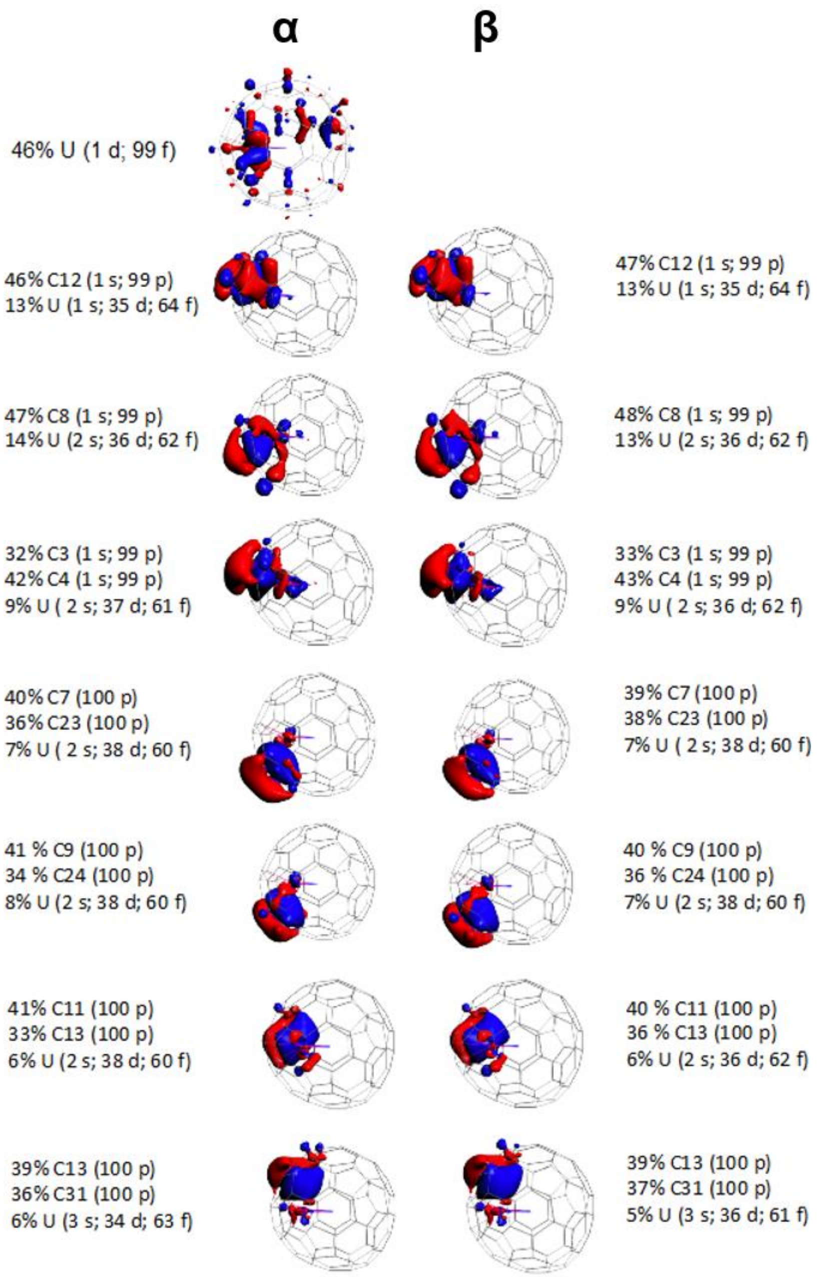

Supplementary Fig. 14. NLMO isosurfaces (±0.03 a.u.) of the carbon cage and atomic orbital %-compositions obtained from a natural bond orbital analysis of the ZORA/DFT/PBE doublet state obtained for UN@C_s(6)-C_{82}. Alpha (α)- and beta (β)-spins are plotted separately.

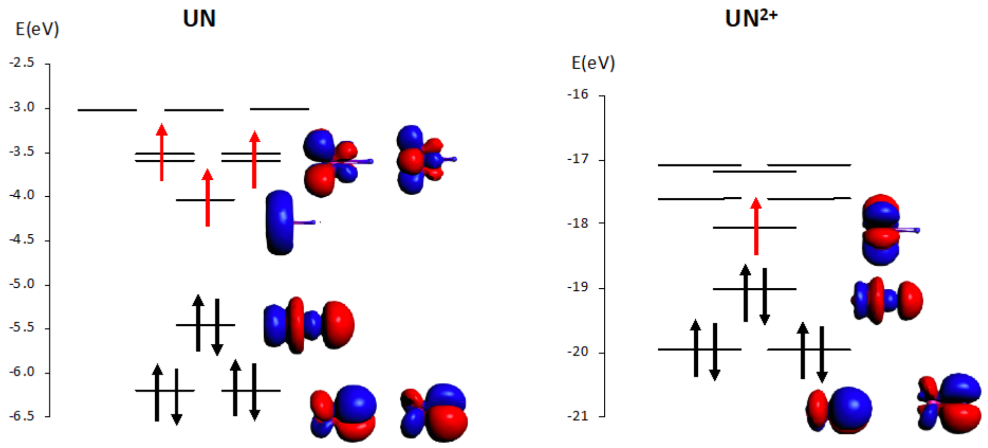

Supplementary Fig. 15. Molecular orbital (MO) diagram obtained with ZORA/PBE/TZP for the ground spin-quartet state of UN and spin-doublet state of UN^{2+}. The singly-occupied molecular orbitals (for α-spin) are drawn in red. MO isosurfaces (±0.04 a.u.) are shown on the side.

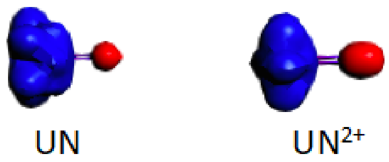

Supplementary Fig. 16. Isosurface (±0.01 a.u.) of the spin density (SD) distribution for the spin-quartet state UN and spin-doublet state of UN^{2+}.

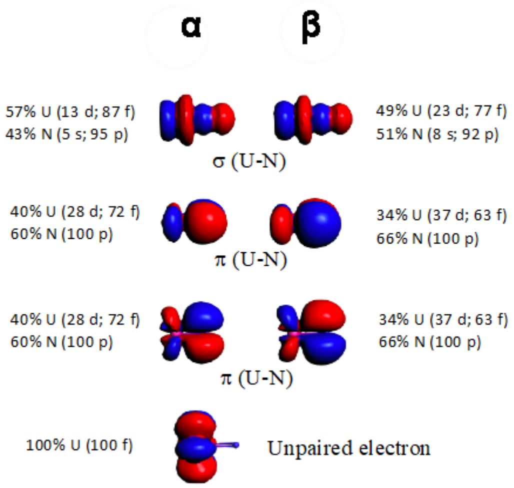

Supplementary Fig. 17. NLMO isosurfaces (±0.03 a.u.) and atomic orbital %-compositions obtained from a natural bond orbital analysis of the ZORA/DFT/PBE doublet state obtained for UN^{2+}. Alpha (α)- and beta (β)-spins are plotted separately.

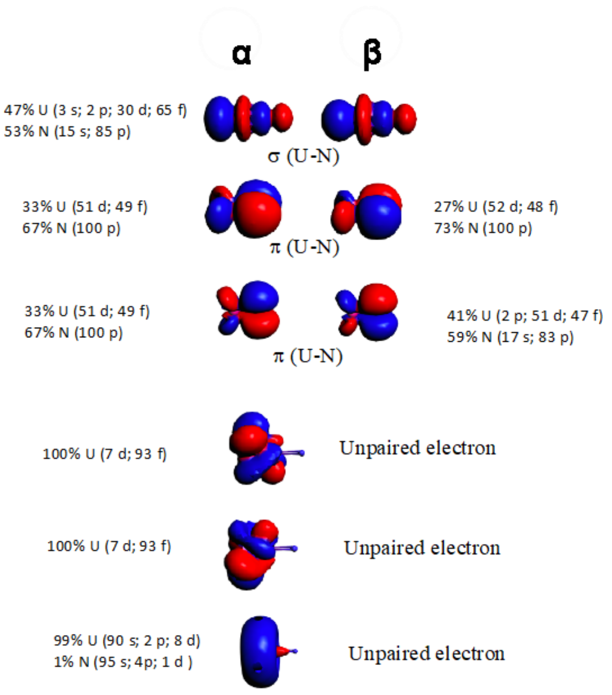

Supplementary Fig. 18. NLMO isosurfaces (±0.03 a.u.) and atomic orbital %-compositions obtained from a natural bond orbital analysis of the ZORA/DFT/PBE quartet state obtained for UN. Alpha (α)- and beta (β)-spins are plotted separately.

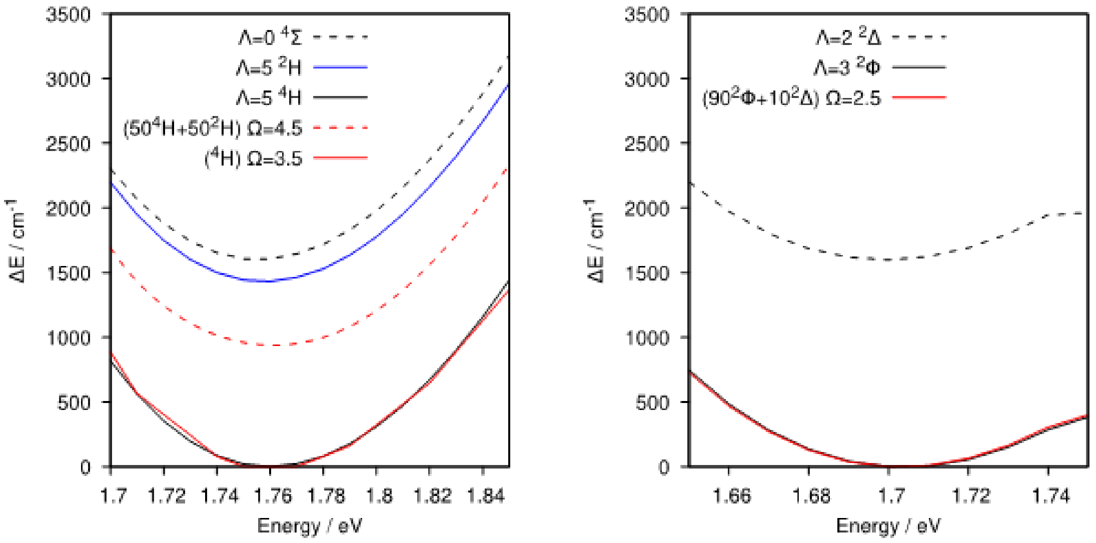

Supplementary Fig. 19. Left panel: XMS-CASPT2(-SO) Potential Energy Surface (PES) scans along the internuclear distance of UN. Right panel: XMS-CASPT2(-SO) Potential Energy Surface (PES) scans along the internuclear distance of UN^{2+}.

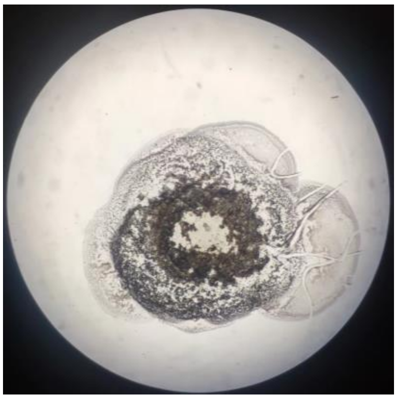

Supplementary Fig. 20. Shape of the sample during Raman testing.

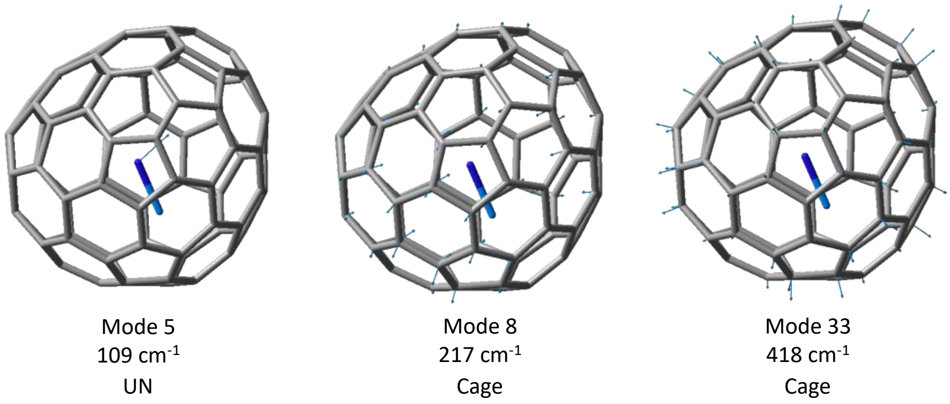

Supplementary Fig. 21. Vibrational normal modes of UN@C_2(5)-C_{82} assigned to the major peaks in the low-energy Raman spectra. For each mode, the wavenumber and arrows indicating the atoms that move are shown.

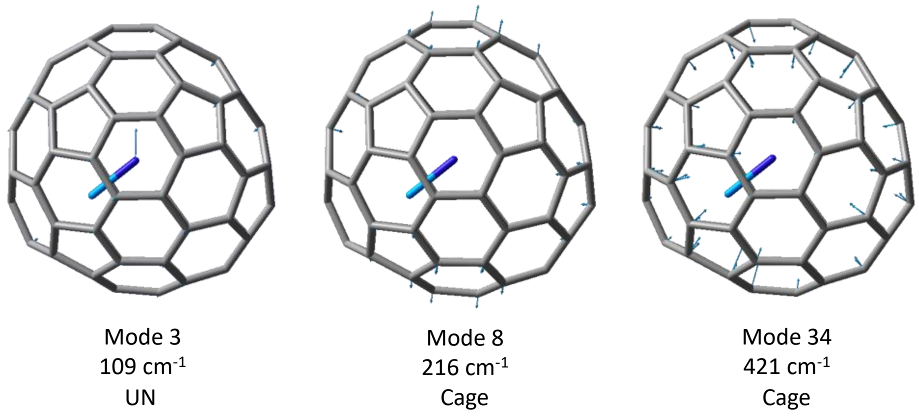

Supplementary Fig. 22. Vibrational normal modes of UN@C_s(6)-C_{82} assigned to the major peaks in the low-energy Raman spectra. For each mode, the wavenumber and arrows indicating the atoms that move are shown.

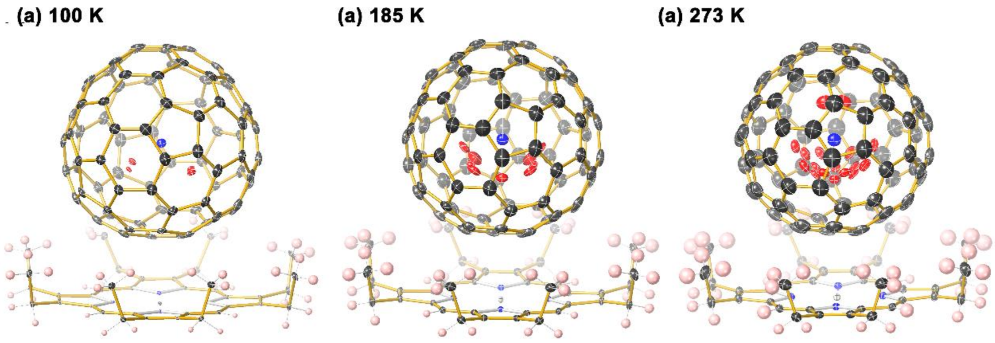

Supplementary Fig. 23. Single-crystal X-ray structure of UN@C_s(6)-C_{82}·[Ni^{II}(OEP)] measured at 100 K(**a**), 185 K(**b**) and 273 K(**c**). Solvent molecules are omitted for clarity. The displacement parameters are shown at the 20% probability level. Color code: black for carbon, red for U, blue for N, pink for H, and grey for Ni.

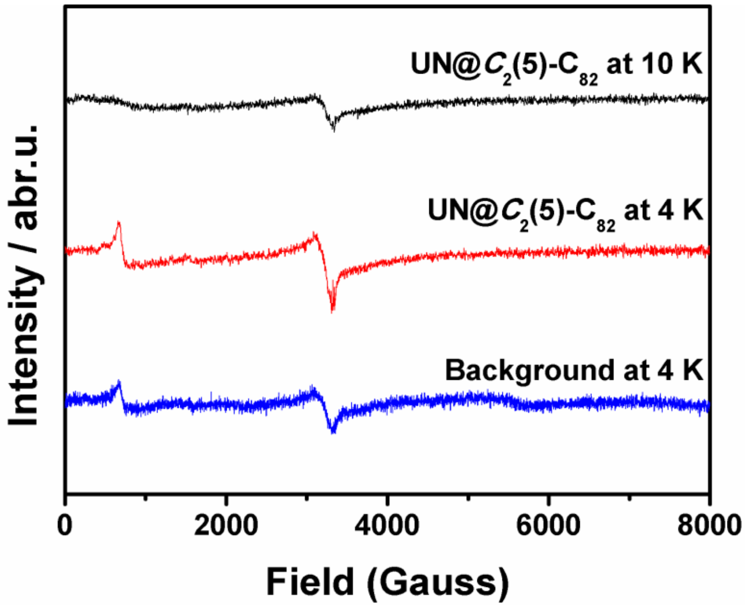

Supplementary Fig. 24. EPR spectrum of UN@C_2(5)-C_{82} at 4K and 10K. Source data are provided as a Source Data file.

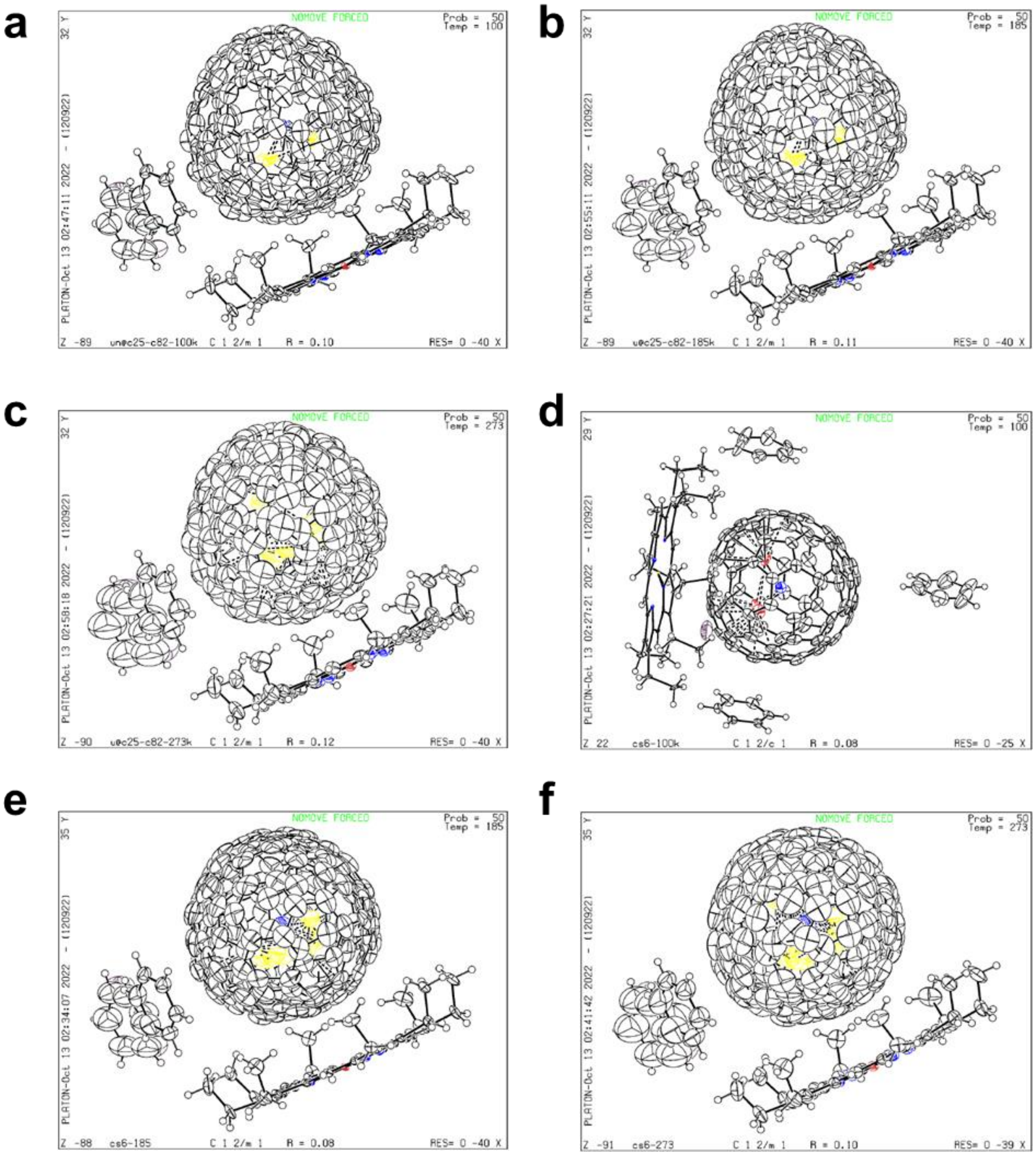

Supplementary Fig. 25. ORTEP-style illustration with probability ellipsoids for **a.** UN@C_2(5)-C_{82}-100K (CCDC no. 2050571), **b.** UN@C_2(5)-C_{82}-185K (CCDC no. 2120708), **c.** UN@C_2(5)-C_{82}-273K (CCDC no. 2120709), **d.** UN@C_s(6)-C_{82}-185K (CCDC no. 2120710), **e.** UN@C_s(6)-C_{82}-185K (CCDC no. 2120939) and **f.** UN@C_s(6)-C_{82}-273K (CCDC no. 2120731). Only one Alert level B in checkcif is "PLAT088_ALERT_3_B Poor Data / Parameter Ratio". The explanation is "The refinement of the cage disorder requires more parameters, resulting in relatively low data/parameter ratio.".

Supplementary Tables

Supplementary Table 1. The distance between U1 and C_{cage} in UN@C_2(5)-C_{82} at 100 K.

Labelling	U1-C54	U1-C53	U1-C72	U1-C55	U1-C32	U1-C34
Length / Å	2.478(15)	2.592(18)	2.646(16)	2.652(17)	2.792(20)	2.861(22)

Supplementary Table 2. The distance between U1 and C_{cage} in UN@C_s(6)-C_{82} at 100 K.

Labelling	U1-C1	U1-C6	U1-C10	U1-C7	U1-C2	U1-C5
Length / Å	2.522(7)	2.503(7)	2.637(7)	2.689(7)	2.785(7)	2.714(7)

Supplementary Table 3. Adiabatic spin-state relative energies (ΔE, kcal·mol^{-1}), U Mulliken Spin Populations (MSP) and structural parameters (distances in Å) for UN@C_{82} isomers 5 and 6. Experimental values in parenthesis.

Isomer	Spin state	ΔE	MSP (U)	U-N	U-C1	U-C2	U-C3	U-C4
UN@ C_2(5)- C_{82}	Doublet	5.3	0.8	1.764 (1.760(20))	2.503 (2.478(15))	2.515 2.592(18)	2.624	2.634
	Quartet	23.2	1.0	1.766	2.502	2.546	2.623	2.631
UN@ C_s(6)- C_{82}	Doublet	0.0	0.5	1.760 (1.760(7))	2.476 (2.503(7))	2.506 (2.522(7))	2.551	2.631
	Quartet	16.6	0.9	1.760	2.514	2.521	2.600	2.673

Supplementary Table 4. Metal site occupancy in UN@C_s(6)-C_{82} as a function of temperatures.

Metal site	100 K	185 K	273 K
U1	0.6442 (1)	0.313 (0.5)	0.205 (0.5)
U2	0.1903 (1)	0.0192 (0.5)	0.0518 (0.5)
U3	0.1087 (1)	0.0630 (0.5)	0.0498 (0.5)
U4	0.0566 (1)	0.082 (0.5)	0.0427 (0.5)
U5		0.0315 (0.5)	0.0422 (0.5)
U6			0.0210 (0.5)
U7			0.0251 (0.5)
U8			0.0246 (0.5)
U9			0.0408 (0.5)
U10			0.0193 (0.5)
U11			0.01091 (0.5)

The occupancy rate of the carbon cage corresponding to the metal site is in the brackets.

Supplementary Table 5. Metal site occupancy in UN@C_2(5)-C_{82} as a function of temperatures.

Metal site	100 K	185 K	273 K
U1	0.312 (0.5)	0.289 (0.5)	0.260 (0.5)
U2	0.188 (0.5)	0.211 (0.5)	0.079 (0.5)
U3			0.055 (0.5)
U4			0.0265 (0.5)
U5			0.0303 (0.5)
U6			0.068 (0.5)
U7			0.0202 (0.5)

The occupancy rate of the carbon cage corresponding to the metal site is in the brackets.

Supplementary Table 6. Crystal data of UN@C_s(6)-C_{82}.

**Crystal**	**UN@C_s(6)-C_{82} at 100K**	**UN@C_s(6)-C_{82} at 185 K**	**UN@C_s(6)-C_{82} at 273 K**
**Formula weight**	1986.26	1986.82	1987.39
**Crystal system**	monoclinic	monoclinic	monoclinic
**Space group**	*C*2/*c*	*C*2/*m*	*C*2/*m*
***a*, Å**	25.3086(13)	25.3712(6)	25.4546(11)
***b*, Å**	14.9637(7)	15.0697(3)	15.2260(6)
***c*, Å**	39.809(2)	19.9969(5)	20.0570(9)
***α*, deg**	90	90	90
***β*, deg**	94.401(2)	94.6860(10)	95.1660(10)
***γ*, deg**	90	90	90
**Volume, Å^3**	15031.6(13)	7620.0(3)	7741.9(6)
Z	8	4	4
***T*, K**	100	185	273
**Radiation (*λ*, Å)**	Synchrotron Radiation (0.82641)	Synchrotron Radiation (0.82641)	Synchrotron Radiation (0.82641)
**Unique data (R_{int})**	14570 (0.0624)	7664 (0.0673)	7772 (0.0473)
**Parameters**	1288	1048	1111
**Restraints**	1082	1383	1516
**Observed data ($I > 2\sigma(I)$)**	13753	7127	7039
**R_1 (observed data)**	0.0764	0.0831	0.0995
**wR_2 (all data)**	0.1961	0.2342	0.2581
**CCDC NO.**	2120710	2120939	2120731

The least occupied U site (0.0566 occupancies with a density of 6.21) in the crystal of UN@C_s(6)-C_{82} is still heavier than a complete carbon atom (with an average density of ~3).

Supplementary Table 7. Crystal data of UN@C_2(5)-C_{82}.

**Crystal**	**UN@C_2(5)-C_{82} at 100K**	**UN@C_2(5)-C_{82} at 185K**	**UN@C_2(5)-C_{82} at 273K**
**Formula weight**	1987.44	1987.46	1987.69
**Crystal system**	monoclinic	monoclinic	monoclinic
**Space group**	*C*2/*m*	*C*2/*m*	*C*2/*m*
***a*, Å**	25.3001(8)	25.3680(6)	25.463(2)
***b*, Å**	14.9553(4)	15.0340(3)	15.1781(13)
***c*, Å**	19.9754(6)	20.0363(4)	20.1103(18)
***α*, deg**	90	90	90
***β*, deg**	94.7540(10)	94.8360(10)	95.028(3)
***γ*, deg**	90	90	90
**Volume, Å^3**	7532.1(4)	7614.3(3)	7742.4(12)
Z	4	4	4
***T*, K**	100	185	273
**Radiation (*λ*, Å)**	Synchrotron Radiation (0.82641)	Synchrotron Radiation (0.82641)	Synchrotron Radiation (0.82641)
**Unique data (R_{int})**	7510 (0.0566)	7600 (0.0488)	7757 (0.0479)
**Parameters**	1029	1029	1068
**Restraints**	1384	1376	1437
**Observed data ($I > 2\sigma(I)$)**	7055	7069	7013
**R_1 (observed data)**	0.1026	0.1065	0.1211
**wR_2 (all data)**	0.2400	0.2718	0.3054
**CCDC NO.**	2050571	2120708	2120709

Supplementary Table 8. Adiabatic spin-state relative energies (ΔE, kcal·mol^{-1}) and U Mulliken Spin Populations (MSP) for UN@C_2(5)-C_{82} and UN@C_s(6)-C_{82} using different functionals: PBE, BP86, PBE0 and B3LYP.

Isomer	Spin state	PBE	MSP	BP86	MSP	PBE0	MSP	B3LYP	MSP
C_2(5)-C_{82}	Doublet	5.3	0.8	4.3	0.9	2.9	1.2	2.4	1.2
	Quartet	23.2	1.0	21.8	1.1	23.5	1.2	22.7	1.2
C_s(6)-C_{82}	Doublet	0.0	0.5	0.0	0.7	0.0	1.2	0.0	1.2
	Quartet	16.6	0.9	15.8	1.0	17.1	1.2	16.1	1.2

Supplementary Table 9. Comparison of the calculated N-U distance (in Å) using different functionals: PBE, BP86, PBE0 and B3LYP, vs. the experimental value for UN@C_2(5)-C_{82} and UN@C_s(6)-C_{82}.

Isomer	Spin state	PBE	BP86	PBE0	B3LYP	Expt.
C_2(5)-C_{82}	Doublet	1.764	1.769	1.744	1.758	1.760(20)
C_s(6)-C_{82}	Doublet	1.760	1.764	1.744	1.759	1.760(7)

Supplementary Table 10. Adiabatic spin-state relative energies (ΔE, kcal·mol^{-1}) and U Mulliken Spin Populations (MSP) for UN@C_2(5)-C_{82} and UN@C_s(6)-C_{82} using different software: ADF vs. G16.

		ADF/PBE/TZP/D3		G16/PBE/6-31G** & SDD/D3	
	Spin state	ΔE	MSP (U)	ΔE	MSP (U)
C_2(5)-C_{82}	Doublet	5.3	0.8	5.5	0.8
	Quartet	23.2	1.0	23.5	1.0
C_s(6)-C_{82}	Doublet	0.0	2.0	0.0	0.5
	Quartet	16.6	2.6	16.4	0.9

Supplementary Table 11. Comparison of the calculated N-U distance (in Å) using ADF vs. G16. The data are compared to the corresponding experimental value for UN@C_2(5)-C_{82} and UN@C_s(6)-C_{82}.

Isomer	Spin state	ADF/PBE/TZP/D3	G16/PBE/6-31G**&SDD/D3	Expt.
C_2(5)-C_{82}	Doublet	1.764	1.755	1.760(20)
C_s(6)-C_{82}	Doublet	1.760	1.750	1.760(7)

Supplementary Table 12. Relative energies (in kcal·mol^{-1}) and structural parameters (distances in Å) of UN, obtained with ZORA/PBE/TZP/D3 for optimized UN@C_2(5)-C_{82} spin-doublet geometries with different orientation of the UN cluster inside the C_2-C_{82} cage.

Orientation	Erel	d(U-N)	d(U-C1 cage)	d(U-C2 cage)	d(U-C3 cage)	d(U-C4 cage)
U1	0.0	1.764	2.634	2.515	2.503	2.624
Ori1	1.7	1.758	2.506	2.514	2.516	2.534
Ori2	2.5	1.763	2.479	2.511	2.596	2.563

Supplementary Table 13. Relative energies (in kcal·mol^{-1}) and structural parameters (distances in Å) of UN, obtained with ZORA/PBE/TZP/D3 for optimized UN@C_s(6)-C_{82} spin-doublet geometries with different orientation of the UN cluster inside the C_s-C_{82} cage.

Orientation	Erel	d(U-N)	d(U-C1 cage)	d(U-C2 cage)	d(U-C3 cage)	d(U-C4 cage)
U1	0.0	1.760	2.476	2.506	2.551	2.631
Ori1	7.6	1.756	2.451	2.518	2.511	2.543
Ori2	4.4	1.753	2.517	2.523	2.545	2.563
Ori3	11.2	1.757	2.504	2.552	2.503	2.552

Supplementary Table 14. U-N distance in UN@C_s(6)-C_{82} as a function of temperatures.

**Metal site**	**100 K / Å**	**185 K / Å**	**273 K / Å**
U1-N1	1.760(7)	1.740(6)	1.774(7)
U2-N1	1.681(7)	1.750(20)	1.717(12)
U3-N1	1.733(7)	1.801(10)	1.683(12)
U4-N1	1.820(9)	1.736(9)	1.756(10)
U5-N1		1.724(13)	1.721(13)
U6-N1			1.750(20)
U7-N1			1.720(20)
U8-N1			1.744(19)
U9-N1			1.714(17)
U10-N1			1.730(30)
U11-N1			1.670(30)

Supplementary Table 15. U-N distance in UN@C_2(5)-C_{82} at 100K.

**Metal site**	**100 K / Å**
U1-N1	1.760(20)
U2-N1	1.750(20)

Supplementary Table 16. Low-energy electronic states of UN at r_{eq} = 1.756 Å from wavefunction calculations.

PT2-SF $^{2S+1}\Lambda$ state	[%] Main Config.	ΔE (cm^{-1})	PT2-SO ([%] main $^{2S+1}\Lambda$) Ω	ΔE (cm^{-1})
Λ = 5, ^{4}H	[92] 5f($\delta^1\phi^1$)7s^1	0	([100] ^{4}H), Ω = 3.5	0
Λ = 5, ^{2}H	[90] 5f($\delta^1\phi^1$)7s^1	1429	([50] ^{2}H + [50] ^{4}H), Ω = 4.5	847
Λ = 0, $^4\Sigma$	[57] 5f($\delta^2\phi^0$)7s^1 + [37] 5f($\delta^0\phi^2$)7s^1	1598	([50] $^4\Sigma$ + [18] $^2\Pi$ + [15] 2$^2\Sigma$ + [10] 1$^2\Sigma$), Ω = 0.5	2595
Λ = 0, 1$^2\Sigma$	[55] 5f($\delta^2\phi^0$)7s^1 + [39] 5f($\delta^0\phi^2$)7s^1	3261	([74] $^4\Sigma$ + [20] $^4\Pi$), Ω = 1.5	5020
Λ = 1, $^4\Pi$	[94] 5f($\delta^1\phi^1$)7s^1	3822	([78] ^{4}H + [22] ^{2}H), Ω = 5.5	5143
Λ = 1, $^2\Pi$	[94] 5f($\delta^1\phi^1$)7s^1	5317	([52] ^{4}H + [48] ^{2}H), Ω = 4.5	5336
Λ = 0, 1$^2\Sigma$	[50] 5f($\delta^2\phi^0$)7s^1 + [45] 5f($\delta^0\phi^2$)7s^1	7037	([52] $^2\Sigma$ + [28] $^4\Pi$ + [12] $^4\Sigma$), Ω = 0.5	5994

Supplementary Table 17. Low-energy electronic states of UN^{2+} at r_{eq} = 1.707 Å from wavefunction calculations.

PT2-SF $^{2S+1}\Lambda$ state	[%] Main Config.	ΔE (cm^{-1})	PT2-SO ([%] main $^{2S+1}\Lambda$) Ω	ΔE (cm^{-1})
Λ = 3, $^2\Phi$	[85] $\pi^4\sigma^2$-5f($\phi^1\delta^0$)	0	([90] $^2\Phi$ + [10] $^2\Delta$), Ω = 2.5	0
Λ = 2, $^2\Delta$	[82] $\pi^4\sigma^2$-5f($\phi^0\delta^1$)	1613	([100] $^2\Delta$), Ω = 1.5	3414
Λ = 5 ^{2}H	[86] $\pi^4\sigma^1$-5f($\phi^1\delta^1$)	6924	([100] ^{4}H), Ω = 3.5	5765
Λ = 0 $^2\Sigma$	[59] $\pi^4\sigma^1$-5f($\phi^0\delta^2$) + [27] $\pi^4\sigma^1$-5f($\phi^2\delta^0$)	8647	([100] $^2\Phi$), Ω = 3.5	6554
Λ = 5 ^{4}H	[94] $\pi^4\sigma^1$-5f($\phi^1\delta^1$)	9473	([59] ^{4}H), Ω = 4.5	7005

Supplementary Table 18. xyz coordinates for the spin-doublet ground state geometry of UN@C_2(5)-C_{82}.

C	-29.950820	-49.383223	51.204911
C	-31.336072	-49.491651	50.860173
C	-31.551608	-50.841936	50.362155
C	-30.287081	-51.521607	50.303358
C	-29.291849	-50.624094	50.836733
C	-28.016146	-50.589755	50.288222
C	-27.348254	-49.321455	50.087689
C	-27.960528	-48.138284	50.483575
C	-29.297951	-48.166688	51.048118
C	-30.012640	-47.016461	50.559680
C	-31.368909	-47.098320	50.137767
C	-32.044420	-48.381397	50.324928
C	-33.144966	-48.697820	49.466997
C	-33.416140	-50.038088	49.035405
C	-32.573826	-51.123572	49.422070
C	-32.336577	-52.155088	48.468984
C	-31.062483	-52.801791	48.390467
C	-29.978788	-52.420232	49.240082
C	-28.625193	-52.392673	48.670667
C	-27.683561	-51.453227	49.182997
C	-26.805691	-50.720704	48.314252
C	-26.653123	-49.374851	48.833599
C	-26.565679	-48.258672	47.970460
C	-27.163563	-46.995806	48.414042
C	-27.833552	-46.971564	49.664489
C	-29.058650	-46.214748	49.830641
C	-29.486417	-45.348366	48.856450
C	-30.884434	-45.260595	48.529839
C	-31.819774	-46.182083	49.079276
C	-32.940282	-46.561231	48.223930
C	-33.594151	-47.809126	48.449985
C	-34.204781	-48.586395	47.375105
C	-34.034925	-49.979932	47.739961
C	-33.727816	-50.981538	46.755854
C	-32.888701	-52.057569	47.133224
C	-31.924378	-52.635038	46.214250
C	-30.840379	-53.138136	47.005267
C	-29.560267	-53.132454	46.472515
C	-28.436824	-52.769056	47.312244
C	-27.478587	-52.110207	46.476974
C	-26.731926	-50.984763	46.932079
C	-26.428534	-49.883812	46.022442
C	-26.359790	-48.516367	46.544644
C	-26.584435	-47.447685	45.645717
C	-27.245206	-46.229020	46.068131
C	-27.572168	-46.005579	47.420171
C	-28.705810	-45.176038	47.659025
C	-29.607581	-44.812260	46.603227
C	-30.942013	-44.835666	47.157199
C	-32.006119	-45.220463	46.351220
C	-33.033331	-46.056711	46.893239
C	-33.569154	-46.862723	45.802682
C	-34.208526	-48.129818	46.006846
C	-33.952833	-49.153248	44.990076
C	-33.665368	-50.531739	45.387080
C	-32.618504	-51.044100	44.536896
C	-31.695281	-52.070356	44.932597
C	-30.353433	-52.048393	44.362073
C	-29.327749	-52.661001	45.134910
C	-27.987135	-52.136864	45.128028
C	-27.628072	-51.152619	44.245441
C	-26.756243	-50.066736	44.652885
C	-27.047240	-48.965754	43.789413
C	-26.930820	-47.665559	44.269459
C	-27.883677	-46.650985	43.876235
C	-28.085502	-45.768551	44.995506
C	-29.357294	-45.177424	45.250845
C	-30.487572	-45.545503	44.389776
C	-31.795829	-45.553697	44.968074
C	-32.766064	-46.552024	44.627176
C	-32.464513	-47.560193	43.667885
C	-33.038614	-48.859533	43.891309
C	-32.244239	-50.022899	43.614914
C	-30.934938	-49.968823	43.026159
C	-29.970935	-50.999462	43.403829
C	-28.594860	-50.649655	43.295594
C	-28.170190	-49.323424	42.939094
C	-29.102848	-48.352595	42.586551
C	-28.952442	-46.984369	43.045303
C	-30.262721	-46.451307	43.312038

C	-31.219443	-47.484414	42.999069	N	-30.146979	-48.776739	46.812675
C	-30.495186	-48.685291	42.609897	U	-31.826818	-48.889506	46.284780

Supplementary Table 19. xyz coordinates for the spin-doublet ground state geometry of UN@C_s(6)-C_{82}.

U	15.942839	8.772680	14.376455	C	19.668339	5.936092	16.280123
N	16.551046	7.290517	13.648367	C	18.580534	5.060990	16.579887
C	15.319412	10.932507	15.414580	C	17.548877	5.589246	17.408132
C	16.589338	10.688103	16.059756	C	16.181951	5.145699	17.261058
C	16.792286	9.646909	17.062362	C	15.856396	4.151582	16.355827
C	15.757382	8.716571	17.395698	C	14.633454	4.218972	15.602564
C	14.511488	8.901085	16.712670	C	13.796470	5.368119	15.628761
C	14.262031	9.998130	15.774566	C	13.045492	5.707707	14.409473
C	13.357060	9.447603	14.765775	C	13.177076	4.855034	13.276254
C	13.419307	9.814134	13.381553	C	13.059922	5.355177	11.932259
C	14.487838	10.691245	13.016680	C	12.723861	6.684371	11.695539
C	15.417689	11.240510	13.999102	C	13.398122	7.432742	10.653286
C	16.707306	11.318518	13.316180	C	14.384204	6.826058	9.882756
C	17.957206	11.151821	13.988959	C	15.590354	7.534431	9.542365
C	17.873607	10.843888	15.384153	C	15.864261	8.838300	10.045619
C	18.820009	9.960005	15.978785	C	17.269377	9.224439	10.253630
C	18.151696	9.217826	17.012944	C	18.277494	8.265301	9.963325
C	18.532338	7.871004	17.331277	C	19.509289	8.206198	10.705177
C	17.506753	6.979502	17.754668	C	19.821287	9.186294	11.628965
C	16.120447	7.402485	17.773814	C	20.483148	8.846187	12.868008
C	15.299175	6.265353	17.415419	C	20.768610	7.489341	13.194490
C	14.150695	6.404386	16.589952	C	20.764667	7.154203	14.582800
C	13.766143	7.750480	16.292666	C	20.390004	5.830905	15.031590
C	13.056817	8.085216	15.101721	C	20.053433	4.855761	14.113720
C	12.671213	7.093255	14.143220	C	18.977174	3.934638	14.394334
C	12.509670	7.559440	12.809719	C	18.233673	4.017213	15.607537
C	12.969245	8.880622	12.421416	C	16.892531	3.556017	15.534365
C	13.601739	8.772637	11.120479	C	16.289077	3.186292	14.288027
C	14.808207	9.470074	10.831878	C	14.886368	3.511697	14.375016
C	15.191521	10.461878	11.788055	C	14.179695	3.817606	13.241209
C	16.553407	10.849647	11.972608	C	14.830090	3.820234	11.943519
C	17.616133	10.255030	11.225194	C	14.075711	4.714341	11.115870
C	18.887403	10.249730	11.863291	C	14.722507	5.432920	10.115293
C	19.048872	10.651815	13.243196	C	16.133426	5.268062	9.927103
C	20.025674	9.773945	13.859278	C	16.633093	6.542564	9.444143
C	19.895364	9.384574	15.221685	C	17.939740	6.902367	9.650496
C	20.315816	8.075003	15.576598	C	18.833735	6.012663	10.350743
C	19.636542	7.320653	16.628389	C	19.833291	6.820938	10.985080

C	20.422595	6.446568	12.221743
C	20.072436	5.150147	12.700307
C	19.027738	4.381314	12.100138
C	18.351896	3.631171	13.145482

C	16.977885	3.317954	13.046111
C	16.240115	3.698767	11.836588
C	16.920749	4.454948	10.783568
C	18.331314	4.821136	10.952220
