# Supplementary material for: A charged diatomic triple-bonded U≡N species trapped in C82 fullerene cages
Source: Nat Commun. 2022 Nov 23;13:7192. doi: 10.1038/s41467-022-34651-5 (PMC9684569; doi:10.1038/s41467-022-34651-5)
Supplement: Supplementary file 3 — Description of Additional Supplementary Files [file 41467_2022_34651_MOESM3_ESM.docx]

Supplementary Data File 1: The cif files of six crystals in this work.
